# Heterophylly: Phenotypic Plasticity of Leaf Shape in Aquatic and Amphibious Plants

**DOI:** 10.3390/plants8100420

**Published:** 2019-10-16

**Authors:** Gaojie Li, Shiqi Hu, Hongwei Hou, Seisuke Kimura

**Affiliations:** 1 The State Key Laboratory of Freshwater Ecology and Biotechnology, The Key Laboratory of Aquatic Biodiversity and Conservation of Chinese Academy of Sciences, Institute of Hydrobiology, Chinese Academy of Sciences, University of Chinese Academy of Sciences, Wuhan 430072, Hubei, China; ligaojie@ihb.ac.cn (G.L.); hushiqi@ihb.ac.cn (S.H.); houhw@ihb.ac.cn (H.H.); 2 Faculty of Life Sciences, Kyoto Sangyo University, Kamigamo-motoyama, Kita-ku, Kyoto-shi, Kyoto 603-8555, Japan; 3 Center for Ecological Evolutionary Developmental Biology, Kyoto Sangyo University, Kamigamo-motoyama, Kita-ku, Kyoto-shi, Kyoto 603-8555, Japan

**Keywords:** heterophylly, submergence, environment, adaptation, molecular mechanisms

## Abstract

Leaves show great diversity in shape, size, and color in nature. Interestingly, many plant species have the ability to alter their leaf shape in response to their surrounding environment. This phenomenon is termed heterophylly, and is thought to be an adaptive feature to environmental heterogeneity in many cases. Heterophylly is widespread among land plants, and is especially dominant in aquatic and amphibious plants. Revealing the mechanisms underlying heterophylly would provide valuable insight into the interaction between environmental conditions and plant development. Here, we review the history and recent progress of research on heterophylly in aquatic and amphibious plants.

## 1. What is Heterophylly?

**Figure 1:**
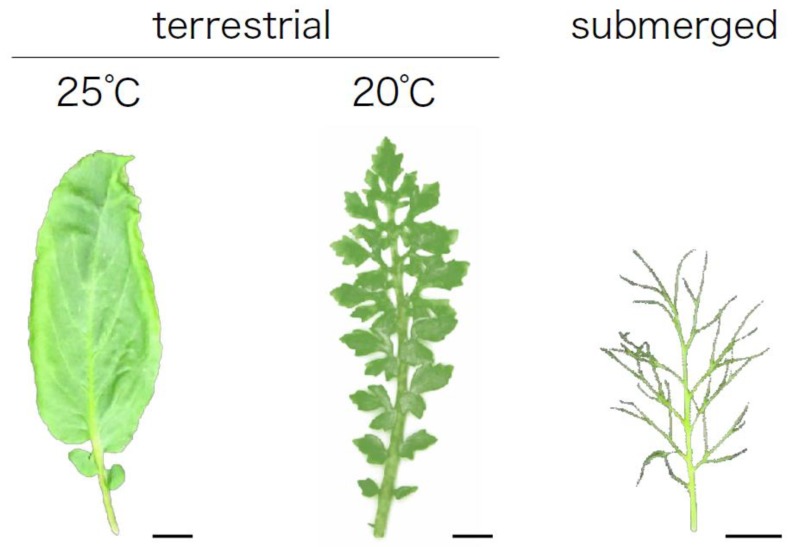
**Heterophylly of amphibious plant, North American lake cress, *Rorippa aquatica* (Brassicaceae).** North American lake cress shows distinct heterophylly between submerged and terrestrial conditions. Leaf shape alternation is also induced by changes in ambient temperature. Bars, 1cm.

Plants display amazing morphological diversity of leaves. The leaves of some plant species can undergo considerable form alteration in response to environmental conditions via a process called heterophylly (Figure 1) [1]. Heterophylly is a type of phenotypic plasticity that is widespread among plants. 

Heterophyllous plants produce dramatic, often abrupt changes in leaf morphology in response to environmental factors [1]. Interestingly, most known examples of heterophylly are found in aquatic and amphibious plants, in which submerged leaves are often dissected compared with simple terrestrial leaves [2] (Table 1). Because these plants are sometimes submerged during flooding, they have evolved to thrive and grow both under water and terrestrial conditions. Such plants often display heterophylly, which is generally regarded as a morphological process allowing adaptation to a capricious environment [2,3]. Leaf shapes are related to their function, as submerged leaves are thin, narrow, and lack cuticles and stomata, whereas terrestrial leaves are thicker, expanded, and cutinized with stomata [2,3]. An example is the narrow leaves of amphibious plants located along riverbanks where flooding always occurs. Narrow leaves are less efficient at absorbing sunlight than those that have wider blades; however, they can withstand the destructive force of water flow. Narrow or deeply serrated/lobed leaves are also present in aquatic plants, and may provide for similar interactions with the surrounding environment, including factors beyond the submerged conditions for mineral nutrient and CO_2_ uptake. These narrow or dissected leaf blade formations are likely an adaptation to underwater conditions. 

The original definition of heterophylly was not strictly linked to the environment, and lacked a clear distinction from other similar processes [1]. However, heterophylly was recently defined as leaf form alteration in response to environmental conditions, unlike heteroblasty and anisophylly [1,4,5,6]. Heteroblasty was described as changes in leaf shape during growth development, but does not include morphological changes induced by environmental factors [1,5]. Similarly, anisophylly is usually coupled with asymmetry and phyllotaxis of leaves and stems, and also does not include morphological changes induced by environmental factors [7]. 

Considering the distinct differences in leaf shape between aerial and submerged conditions, elucidating the mechanisms underlying heterophylly in aquatic and amphibious plants would provide valuable insight into the interaction between the environment and plant development. 

## 2. History of Research on Heterophylly

Heterophylly is observed in many evolutionary diverse aquatic and amphibious plant species including those belonging to the Nymphaeales, Ranunculales, Saxifragales, Myrtales, Brassicales, Lamiales, and many other orders [8,9,10,11,12,13,14,15,16] (Table 1). There are remarkable morphological differences in these heterophyllous plants between submerged and terrestrial environments. Leaves under submerged conditions tend to have a thin, filamentous, or linear shape, degraded vascular structure, and the leaves usually lack stomata cells, which means plants must directly absorb nutrients and exchange gas from water [2,3,17,18]. Here, we review the existing knowledge of heterophylly in different species, with a particular focus on the role of phytohormones and environmental factors in regulating heterophylly, to gain valuable insight into this phenomenon.

Multiple phytohormones are involved in the regulation of heterophylly. For example, gibberellic acid (GA) can induce floating plants to develop aquatic leaves in the two-headed water starwort, *Callitriche heterophylla* (Callitrichaceae), and pond water starwort, *Callitriche stagnalis*, while abscisic acid (ABA) can induce submerged plants to grow floating leaves in the two-headed water starwort [11,20,21]. Kane and Albert (1987b) [17] also found that ABA can induce the aerial leaf morphology and vasculature in submerged common mare’s tails, *Hippuris vulgaris* (Hippuridaceae). Kane and Albert (1989) [10] later studied nine plant species in the genera *Myriophyllum* or *Proserpinaca* (Haloragaceae), and found that ABA plays a common role in the regulation of leaf development, induction of stomata development, increased cuticularization, and reduced leaf and epidermal cell length in these heterophyllous plants. In 1990, ABA was also observed to induce the terrestrial leaf phenotype in the yellow water buttercup, *Ranunculus flabellaris* (Ranunculaceae). Later, Lin and Yang (1999) [12] studied European water clover, *Marsilea quadrifolia* (Marsileaceae), and found that ABA could induce its terrestrial phenotype under submerged conditions.

In addition to ABA, other phytohormones regulate this process. Kane and Albert (1987a) [9] studied the functions of two phytohormones, GA and ABA, and found that both regulate the heterophylly of marsh mermaid-weed, and these two phytohormones were regulated by environmental stimuli, including photoperiod and water stress. In Piedmont primrose-willow, *Ludwigia arcuata* (Onagraceae), ABA and ethylene have antagonistic effects on the regulation of heterophylly. Ethylene induced submerged-type leaves in terrestrial conditions, while ABA induced aerial phenotypes under submerged conditions [14,29]. Furthermore, ethylene affects the frequency and direction of cell division, and low temperature also enhances the effects of ethylene in Piedmont primrose-willow [6,30]. Recent studies on threadleaf crowfoot, *Ranunculus trichophyllus* (Ranunculaceae), showed that ABA and ethylene signaling are the key regulatory pathways for heterophylly in this species. Aquatic leaves have higher levels of ethylene and lower levels of ABA than terrestrial leaves [43].

Research on the regulation of heterophylly by environmental factors was first carried out in 1902 by McCallum (1902); the author found that marsh mermaid-weed, *Proserpinaca palustris* (Haloragidaceae), has a broad, serrated leaf shape, and well-developed vascular system when leaves are not submerged, but has a dissected, thread leaf shape, lacks a xylem, and has a weak phloem under submerged conditions. McCallum (1902) [37] tested the effects of environmental factors, such as light, nutrition, temperature, humidity, salinity, and the concentration of CO_2_ and O_2_, and found that humidity can significantly change the leaf shape of common mermaid-weed.

In the past several decades, a large amount of experimental work has been published on the effects of environmental conditions on the leaf shapes of heterophyllous plants [20,21,22,24,34,41]. For example, 30% artificial sea water (which results in high osmotic stress) resulted in the formation of aerial leaves in narrowleaf water starwort, *Callitriche intermedia*, grown under submerged conditions [22]. Furthermore, high temperature, mannitol, or artificial sea water can induce submerged plants to grow floating leaves in the two-headed water starwort [11,20,21].

The leaf form of common mare’s tail is affected by light levels and the osmotic pressures in the environment in which it grows, as high light intensity or high concentrations of artificial sea water result in leaves resembling aerial ones in this plant [25]. In addition, Bodkin et al. (1980) [24] also found that a lower red light (wave length 660 nm)/far red light (wave length 730 nm) ratio (R/FR ratios) led to the growth of terrestrial leaves in common mare’s tail.

Johnson (1967) [42] found that yellow water buttercups grown under different conditions have two typical heterophyllic leaves, deep lobed aquatic leaves and shallow lobed terrestrial leaves. According to Johnson [42], low temperature can induce deep lobed leaves in terrestrial plants, as there is a limited exchange of gases in low temperature or aquatic environments, and therefore the increase in this specific surface area is intended to accommodate the diffusion of gases. Later, Bristow (1969) analyzed the effect of CO_2_ on yellow water buttercups, and found that 5% CO_2_ induced the formation of aquatic leaves in terrestrial plants [33]. Furthermore, Cook (1969) [40] found that the divided leaf shape of white water crowfoot, *Ranunculus aquatilis,* can be induced by short photoperiods under terrestrial or submerged conditions, but this leaf shape developed only under submerged conditions with long photoperiods.

Bristow and Looi (1968) [31] tested the effects of CO_2_ in the regulation of heterophylly, and found that higher CO_2_ concentrations can induce aquatic phenotypes in hairy water clover, *Marsilea vestita*. Lin and Yang (1999) [12] studied European water clover and found that blue light could induce a terrestrial phenotype under submerged conditions [12]. A study on yellow water lily, *Nuphar variegata* (Nymphaeaceae), also demonstrated that concentrations of CO_2,_ sediment type, and water depth can all affect the leaf morphogenesis of this species [34]. In addition, a recent study showed that cold conditions and hypoxia can induce aquatic leaf shape formation in threadleaf crowfoot [43].

These studies indicate that environmental factors such as light, nutrition, temperature, humidity, salinity, concentrations of CO_2_ and O_2_, and phytohormones including GA, ABA, and ethylene all are involved in the heterophyllic process. Furthermore, these factors trigger multiple intracellular mechanisms that control heterophylly. 

## 3. Environmental Factors that Induce Heterophylly

Multiple factors such as CO_2_ concentration, light intensity and quality, temperature, osmotic potential, and mechanical forces are involved in the submergence response. Here we review how these environmental factors affect heterophylly. 

### 3.1. CO_2_

The CO_2_ concentration of water is much higher than that of the air, but the rate of diffusion of CO_2_ in water is much slower. Plants may require CO_2_ at a higher rate than is available given the slow rate of diffusion under aquatic conditions. In a study on marsh mermaid-weed, plants were grown under water in vessels containing different CO_2_ concentrations. A submerged leaf shape was produced in every case, indicating that, at least in the absence of CO_2_, the water form of marsh mermaid-weed could not be produced [37]. In 1968, a study of an amphibious plant, hairy water clover, was published [31]. When supplied with increased concentrations of CO_2_ in air, this plant exhibited many leaf characteristics of the water form, such as the orientation of leaf laminae and the shape of epidermal cells [31]. A year later, another two heterophyllous amphibious species, yellow water buttercup and red stemmed parrot feather, *Myriophyllum brasiliense* (Haloragaceae)*,* were found to develop aquatic leaf morphology when grown on solid substrate with 5% CO_2_ in air, while terrestrial plants grown with 0.03% CO_2_ in air still developed terrestrial leaf morphology, and submerged plants developed an intermediate leaf morphology [33]. These results were similar to those obtained with hairy water clover. In addition, high CO_2_ concentrations favored the development of submerged leaf traits over floating leaves in yellow water lily [34]. Thus, these results suggest that, during plant development, CO_2_ sensing pathways are critical for the acquisition of heterophylly, although it may be HCO_3_^−^ (produced by CO_2_ reacting with water) that is the key factor that induces heterophylly [47].

### 3.2. Light Intensity

McCallum (1902) [37] used many aquatic plants to conduct experiments under different light conditions. The results uniformly indicated that light had no effect on the characteristics of developing shoots [37]. However, studies on plants in the genus *Hippuris* demonstrated that leaf shape was in some way affected by light. Broad and round aerial-type leaves developed under a light intensity of 194 μmol photons m^−2^ s^−1^, while aquatic leaves were produced at 97 μmol photons m^−2^ s^−1^ illumination [25]. Experiments on arrowhead, *Sagittaria sagittifolia* (Alismataceae), and *Alisma graminifolium* (Alismataceae) also indicated that absence of light inhibited the formation of aerial leaves, but this effect may not be directly due to the darkness and may be due to a state of inadequate nutrition instead [19]. 

With the rise of sequencing technology, transcriptome analysis has indicated that light intensity affects leaf form alterations in North American lake cress, *Rorippa aquatica* (Brassicaceae) [44,45]. In North American lake cress, dissected leaves with deep biserrated leaflets developed under higher light intensity (90 μmol photons m^−2^ s^−1^) whereas dissected leaves with a relatively smooth margin developed when exposed to lower light intensity (15 μmol photons m^−2^ s^−1^).

### 3.3. Light Quality

Light quality can also be effective in inducing heterophylly. Lin and Yang (1999) [12] reported that blue light can independently induce the transition from submerged type leaves (divided, oblanceolate leaflets, expanded in the plane of the petiole) to aerial type leaves (resembling a four-leaf clover, with quadrifid lamina expanded at an angle to the petiole) in European water clover [12]. Continuous far-red light can cause the fern hairy water clover to develop as its land form (short rhizome and long petioles) instead of the water form (long rhizome and short petioles) [32]. A study on common mare’s tail indicated that when R/FR ratios exceeded a critical range, aerial leaf formation (thick cuticle, irregular rounded cells, and numerous stomata) was inhibited, and only submerged-type leaves (thin cuticle, long thin cells, and no stomata) were formed [24]. 

### 3.4. Light Photoperiod

The photoperiod controls many developmental responses in plants. The response to the photoperiod enables developmental events to be scheduled to meet with particular environmental conditions [48]. Much progress has been made towards understanding the molecular mechanisms involved in the plant response to photoperiod, including those that govern the changes in leaf morphology. It has been shown that short days tend to promote the formation of submerged leaves (highly dissected), and long days promote the formation of aerial-type leaves (less dissected or simply trilobed) in marsh mermaid-weed [38] and intermediate mermaid-weed, *Proserpinaca intermedia* [39]. In addition, another species in the genus *Ranunculus*, white water crowfoot, also developed submerged divided-type leaves (which are composed of long, cylindrical, capillary segments) under short photoperiods, whether under terrestrial or submerged conditions [40]. However, research on variegated pond lilies suggests that the photoperiod only has a mild impact on this leaf morphology alteration [38]. In this case, alternative regulatory mechanisms may exist, such as those involving variation of energy or photosynthetically active radiation.

### 3.5. Temperature

In the past few decades, the effects of temperature on the determination of leaf form have been studied in many heterophyllous species. As described, low temperature induces the formation of submerged-type leaves on terrestrial shoots of yellow water buttercup [42], threadleaf crowfoot [43], marsh mermaid-weed [38], and intermediate mermaid-weed [39]. However, Cook (1968) [40] found that temperatures (between 6 and 20 °C) had almost no effect on the determination of leaf shape formation in white water crowfoot. Temperature only altered some morphological features of the divided leaves, such as the number of dichotomous branches and length of the petiole [40]. Moreover, high temperatures have been reported to induce aerial-type leaves on submerged shoots in aquatic plants, including in the two-headed starwort [11,21], narrowleaf water starwort [23], and common mare’s tail [24]. A study on model plant Piedmont primrose-willow identified temperature as the most crucial factor for forming extremely elongated submerged leaves and elongated epidermal cells [30]. In 2014, researchers proposed North American lake cress as a model plant for the study of heterophylly, since it was found to have a temperature-dependent development of heterophylly. Pinnately dissected leaves developed at lower temperatures under both terrestrial and submerged conditions, indicating a common molecular mechanism of leaf morphogenesis under both of these conditions [45].

### 3.6. Osmotic Stress

Osmotic stress is caused by drought, salinity, or cold stress; all of these factors have had a great impact on plant evolution [49]. Osmotic stress affects both the growth and development of plants, and in particular reduces the productivity of crop plants and affects leaf size and internode length [50,51]. It also involves ABA, ethylene, and GA signaling pathways [52,53,54]. Several heterophyllous aquatic or amphibious plants undergo morphological changes in solutions of various osmotic pressures, including *Marsilea* species, and narrowleaf water starwort [22,23,25]. Leaves of these plants resemble aerial-type leaves when grown in solutions of high osmotic concentration. In common mare’s tail, osmotic stress triggered aerial leaf development, supporting the hypothesis that osmotic stress causes submerged shoots to produce endogenous ABA, which in turn induces the formation of aerial-type leaves [5]. In a study of narrowleaf water starwort, it was suggested that increases in osmotic pressure might directly affect the primordial leaf form through turgor changes that activate an intracellular signaling cascade. A more likely hypothesis is that leaf form alteration was mediated through an auxin-dependent mechanism that responds to fluctuations in the turgor pressure of leaf cells [23]. 

### 3.7. Water Depth

For aquatic plants, water depth is a complex but key factor involved in heterophylly [55]. Many factors change with water depth, including light, pressure, temperature, and CO_2_. It has been demonstrated that light availability decreases with increasing depth. For instance, areas with a water depth of 1 m in the River Rhine have a light transmission below 1% [56]. Thus, heterophylly in aquatic plants is thought to correlate with changes in water depth. A study on *Hippuris* plants implied that in deeper water of the Great Whale River, typical long and flaccid aquatic leaves developed on stems. However, in the shallower permanent pools with depths of 12–18 inches, leaves more commonly displayed the characteristics of aerial-type leaves (thick outer walls, more lateral veins, and mesophyll) [25]. This is consistent with the results in variegated pond lilies, which indicate that shallower depths stimulate the development of floating leaves [34]. The ideas of the classic pattern of zonation also indicate that plants with floating leaves are typically dominant in shallower water, whereas submerged macrophytes are typically dominant in deeper water [55,57].

### 3.8. Water Flow

Aquatic plants grown in streams have various constraints linked to the water flow and adopt strategies to prevent damage due to water pressure and other hydrodynamic forces [58]. As an example, the architecture traits of the modules of *Potamogeton alpinus* (Potamogetonaceae) from slow- and fast-flowing streams have been studied [59]. It was found that in fast-flowing waters, the presence of floating leaves stabilized the vertical position of stem and caused elongation of submerged leaves; together with a reduction in shoot diameter, this reduced the pressure of the water. In addition, plants grown in fast-flowing conditions are more resistant to stretching than others grown in standing waters, which means that stems from fast-flowing conditions are more elastic and therefore less prone to damage by stretching forces [59,60].

As described above, submersion is not the only factor that regulates heterophylly, and other environmental factors also play a role [35,61,62]. The relationship between these factors and the molecular mechanism of leaf phenotype regulation needs to be further examined.

## 4. Molecular Basis for Heterophylly

Heterophylly is an eye-catching phenomenon that has attracted many researchers, and has been studied for a long time. Although much is known about the physiological and cytological aspects of heterophylly, the underlying mechanism regulating the process is largely unknown at the gene level. This is due to the difficulties in performing genetic, genomic, and transcriptomic analyses, because most plants that show significant heterophylly are non-model plant species, and DNA sequence information on these species is limited. However, the recent advent of next-generation sequencing technology has facilitated the analysis of genomes and transcriptomes in non-model plant species. Heterophylly is not dependent on changes in the genome sequence, but is induced by changes in the expression levels and patterns of genes involved in leaf development and environmental responses [63]. Thus, transcriptome analysis is a useful method to elucidate the mechanism of heterophylly at the gene level. In fact, several transcriptome studies have been conducted on multiple heterophyllous plant species. In this section, we summarize the recent progress regarding our understanding of the molecular basis for heterophylly.

### 4.1. North American Lake Cress 

North American lake cress is a perennial amphibious plant whose habitat includes the bays of lakes, ponds, and streams in North America. In nature, the cress shows distinct heterophylly between submerged and terrestrial conditions (Figure 1). The plant develops pinnately dissected leaves with needle-like leaf blades under submerged conditions, while it forms simplified leaves with serrated margins under terrestrial conditions. Interestingly leaf shape alternation is induced by changes in ambient temperature [44,45]. Lower temperatures result in more dissected leaves, which resemble submerged leaves, and higher temperatures simplify the leaf shape. In this cress, the expression levels of *KNOTTED1-LIKE HOMEOBOX* (*KNOX1*) orthologs, which are involved in leaf shape determination in many plant species, changed in response to change of ambient temperature. Furthermore, the accumulation of GA, which is regulated by KNOX1, changed in the leaf primordia. Thus, the regulation of GA levels via KNOX1 is involved in regulating heterophylly in North American lake cress [44,45]. Nakayama et al. (2014b) [45] performed RNA-seq analysis to understand global transcriptional alterations associated with heterophylly induced by temperature change. Interestingly, the genes upregulated in the dissected leaf condition (i.e., those formed under high temperature conditions) also overlapped with those that respond to changes in high light intensity, suggesting that light intensity affects the leaf morphology of this plant. Indeed, under higher light conditions intensity (90 μmol photons m^−2^ s^−1^), dissected leaves with deeply serrated leaflets developed, whereas under lower light conditions intensity (15 μmol photons m^−2^ s^−1^), dissected leaves with a relatively smooth margin developed. Therefore, temperature and light intensity may affect leaf form through a common developmental mechanism. Indeed, plant photoreceptors such as phytochromes are involved in temperature sensing [64,65,66], leading to the hypothesis that light sensing mechanisms might be involved in regulating heterophylly in North American lake cress. 

### 4.2. Threadleaf Crowfoot

Threadleaf crowfoot is also an amphibious plant that shows heterophylly. This plant develops radialized leaves under submerged conditions, but produces flattened broad leaves under aerial conditions. Kim et al (2018) [43] analyzed this plant to understand the molecular basis behind heterophylly. Transcriptome analysis demonstrated that two phytohormones, ethylene and ABA, are involved in regulating heterophylly. Indeed, aquatic leaves produced higher levels of ethylene and lower levels of ABA compared with terrestrial leaves. In submerged leaves, accumulation of ethylene increased the expression of *EIN3* (*ETHYLENE INSENSITIVE3*), an ethylene signaling transducer. The *EIN3*-mediated pathway induced the overproduction of abaxial genes, *KANADI* orthologs, which are implicated in the generation of radialized leaves. The overproduction of *KANADI* orthologs suppressed the expression of *STOMAGEN* and *VASCULAR-RELATD NAC-DOMAIN7* (*VND7*), resulting in lack of stomata and reduced vessel development in submerged leaves. In contrast, ABA activated the expression of adaxial genes, HD-ZIPIII orthologs, which increased *STOMAGEN* and *VND7* under terrestrial conditions. Such responses were not observed in the closely related species cursed buttercup, *Ranunculus sceleratus,* which does not show heterophylly. These results clearly indicate that acquisition of this ABA/ethylene signaling cascade is a key step for evolutionary adaptation to aquatic environments.

### 4.3. Potamogeton octandrus

*P. octandrus* is a perennial aquatic heterophyllous herb found in slow moving fresh water [36]. The floating leaves of this plant are ovate and flat, and submerged leaves are narrow and long. The leaf shape is not only affected by environmental conditions, but also by the developmental stage. In the early stage of development, only submerged type leaves are formed. When the tops of stems reach the water surface, they start producing both floating and submerged leaves. To investigate the molecular basis for this heterophylly, transcriptome analyses of submerged and floating leaves at different developmental points were performed [36]. In total, 6822 differentially expressed genes (DEGs) were identified in 81,103 unigenes. KEGG pathway enrichment analysis demonstrated that many of the DEGs could be classified in the “plant hormone signal transduction” category. Indeed, endogenous levels of hormones such as ABA, cytokinin, GA, and auxin changed between conditions, suggesting that phytohormones play important roles in regulating heterophylly [36,67]. In many heterophyllous plants, stomata and cuticle development are suppressed in submerged leaves. In *P. octandrus*, there are many genes related to stomata and cuticle development in DEGs. Elucidating their precise roles in heterophylly will provide more information on the mechanisms regulating this important process [36]. 

### 4.4. Water-Wisteria, Hygrophila Difformis (Acanthaceae)

Water-Wisteria is an amphibious plant belonging to the *Hygrophila* genus which contains almost 90 species. It is a fast-growing plant that has either simple leaves or highly lobed leaves, depending on the environment. Their leaf shape responds to phytohormones (such as ABA, ethylene, and GA) and environmental factors (such as humidity and temperature). Furthermore, it is easily vegetatively propagated, and can be easily transformed by *Agrobacterium tumefaciens*. Analysis of *KNOX1* of Water-Wisteria (Hd*STM* and Hd*BP*) has revealed that the expression of *KNOX1* orthologs are higher under submerged conditions than under terrestrial conditions. This result is consistent with the pattern of *KNOX1* expression in North American Lake Cress [15,45]. These characteristics suggest that Water-Wisteria is also a good model plant to study heterophylly [26]. Recently, Horiguchi et al. (2019) have analyzed the photosynthetic ability of aerial and submerged leaves of Water-Wisteria and found that this plant acclimates to a submerged environment by developing submerged leaves, and that ethylene is important for this acclimation [27]. 

## 5. Future Perspectives for Studies on Heterophylly

The mechanisms underlying heterophylly remain largely unknown. On the basis of this review, we would like to suggest some of the topics that should be included in future research in this field. 

### 5.1. Sensing Mechanisms for Submergence

Heterophylly of aquatic and amphibious plants is induced by submergence, but it is still unknown how plants perceive submergence. In deepwater rice, *Oryza sativa* (Gramineae), accumulation of ethylene in the tissues during submergence has been shown to induce the elongation of internodes [68]. Piedmont primrose-willow is a well-studied aquatic plant that shows heterophylly and ethylene is involved in leaf shape determination in this plant [7,14,29,30]. Thus, ethylene accumulation is thought to act as a signal for submergence. ABA is known as a stress hormone, and is upregulated under drought conditions, where it functions as a central regulator and integrator of the changes in stomatal behavior, including sensitivity, elicited by external signals [69]. ABA treatment induces the formation of aerial leaves in Piedmont primrose-willow and longleaf pondweed, *Potamogeton nodosus*, which exhibit distinct heterophylly between floating and submerged leaves [4]. Both ethylene and ABA are likely used as signals in the submergence response.

North American lake cress is another plant that can change its leaf shape in response to submergence and changes in temperature and light intensity [15,44,45]. Furthermore, heterophylly in common mare’s tail and *Rotala hippuris* (Lythraceae) is controlled by the ratio of red to far-red light intensity (R/FR) [46]. These results imply direct phytochrome control of the reversible transitions between different types of leaves, because water (and especially deep water) specifically absorbs longer wavelengths light, such as far-red (FR) light [70]. Recently, it was shown that phytochrome is also involved in temperature sensing in plants [64,65,66], which also coincides with the fact that some heterophyllous plants, such as North American lake cress, change leaf shape in response to temperature change. In conclusion, it appears that phytochrome responsiveness is important for the induction of heterophylly in aquatic plants adapted to deep water. 

### 5.2. Epigenetic Regulation of Heterophylly

Heterophylly is induced by changes in gene expression in response to environmental conditions. Therefore, it is critical to understand how gene expression is regulated during the process. Phenotypic plasticity in traits such as flowering is controlled by epigenetic regulation in response to environmental conditions. The ability of plants to respond to environmental changes by epigenetic modifications may play an important role in regulating gene expression in heterophylly [71]. Common holly, *Ilex aquifolium* (Aquifoliaceae), is a heterophyllous tree species that shows two types of leaves, prickly and nonprickly [28]. Interestingly, the production of prickly leaves is induced by mammalian browsing, most likely as a protective response to prevent herbivory [28]. A methylation-sensitive amplified polymorphism (MSAP) analysis demonstrated that DNA methylation profiles are different between prickly and nonprickly leaves, suggesting a correlation between epigenetic status and leaf shapes [28]. It would be interesting to evaluate whether epigenetic regulation is also involved in regulating heterophylly in other species, including aquatic and amphibious plants. 

### 5.3. Adaptive Significance of Heterophylly

In addition to the plasticity of leaves in response to different environments, the relationship between leaf shape and leaf function remains unclear. Leaves are the main photosynthetic organs of plants and have developed numerous physiological, biomechanical, and cellular adaptations to fulfill this function. Thus, one hypothesis is that leaf dissection can increase photosynthesis under particular conditions. Baker-Brosh and Peet (1997) demonstrated that lobed leaves of temperate trees are critical for early season photosynthesis, because this type of leaf can incorporate more CO_2_ [72]. Recently, studies of tomato and cotton revealed that the expression level of specific genes was higher during the development of more complex leaves, which is a possible explanation for the increase in photosynthesis efficiency and fruit sugar content [73,74,75]. Another explanation is that leaf dissection could modulate leaf temperature. One study found that sun leaves (near the top and on the southern sides of trees) of large oaks tend to be more dissected than shade leaves, as leaf dissection could regulate thermal exchanges between leaves and the surrounding environment [76,77]. However, few studies regarding the links between leaf morphology and functions have focused on aquatic plants [36,67]. In addition, the adaptive significance of heterophylly in response to environmental heterogeneity remains unclear. Further studies will be required to address these questions.

### 5.4. Evolution of Heterophylly

Environmental conditions surrounding organisms are not constant, and can vary even from one minute to the next. Phenotypic plasticity, including heterophylly, may play an important role in the adaptation to such fluctuating environments. Acquiring the ability to respond to environmental changes is thought to be especially important for plants, because of their sessile lifestyle. Heterophylly is likely an adaptive feature for aquatic and water’s edge environments. The process has evolved multiple times during plant evolution, and it is perceived to be an adaptive mechanism that allows plants to respond to the changeable environment [3,26,27,28,29,36,46,62,63,64,65,66,67,68,69,70,71,72,73,74,75,76,77,78,79]. Thus, heterophylly is a good example of convergent evolution. Recent progress in research on the molecular basis underlying heterophylly has highlighted the need for a comparative genomics and transcriptome approach to study this important process between plant species. Such comparative approaches will shed light on the evolutionary background of heterophylly in the near future. 

## Figures and Tables

**Table 1:** Representative heterophyllous plants and treatments that can induce heterophylly.

Species	Family	Common Name	Treatments	References
*Alisma graminifolium*	Alismataceae	-	light	[19]
*Callitriche heterophylla*	Callitrichaceae	two-headed water-starwort	ABA, GA, temperature, osmotic stress	[11,20,21]
*Callitriche intermedia*	Callitrichaceae	narrowleaf water-starwort	osmotic stress, temperature	[22,23]
*Callitriche stagnalis*	Callitrichaceae	pond water starwort	GA	[20,21]
*Hippuris vulgaris*	Hippuridaceae	common mare’s tail	ABA, temperature, light intensity, R/FR ratio, osmotic stress	[5,9,24,25]
*Hygrophila difformis*	Acanthaceae	Water-Wisteria	ABA, ethylene, GA, humidity, temperature	[26,27]
*Ilex aquifolium*	Aquifoliaceae	Common holly	mammalian browsing	[28]
*Ludwigia arcuata*	Onagraceae	piedmont primrose-willow	ABA, ethylene, temperature	[6,14,29,30]
*Marsilea quadrifolia*	Marsileaceae	European water clover	ABA, blue light, CO_2_	[12,13]
*Marsilea vestita*	Marsileaceae	hairy water clover	CO_2_, light intnsity, light quality	[31,32]
*Myriophyllum brasiliense*	Haloragaceae	red stemmed parrot feather	CO_2_	[33]
*Nuphar variegate*	Nymphaeaceae	yellow water lily	CO_2_, sediment type and water depth	[34]
*Nuphar lutea*	Nymphaeaceae	yellow pond-lily	water depth	[16]
*Potamogeton nodosus*	Potamogetonaceae	longleaf pondweed	ABA	[4]
*Potamogeton alpinus*	Potamogetonaceae	-	water flow	[35]
*Potamogeton octandrus*	Potamogetonaceae	-	submerged or floating condition, development	[36]
*Proserpinaca palustris*	Haloragidaceae	marsh mermaid-weed	ABA, GA, light intensity, humidity, osmotic stress, photoperiod	[17,37,38]
*Proserpinaca intermedia*	Haloragidaceae	intermediate mermaid-weed	photoperiod	[39]
*Ranunculus aquatilis*	Ranunculaceae	water crowfoot	photoperiod	[40]
*Ranunculus flabellaris*	Ranunculaceae	yellow water buttercup	ABA, temperature, CO_2_	[33,41,42]
*Ranunculus trichophyllus*	Ranunculaceae	threadleaf crowfoot	ABA, ethylene, temperature, hypoxia	[43]
*Rorippa aquatica*	Brassicaceae	North American lake cress	GA, ethylene, temperature, light intensity	[15,44,45]
*Rotala hippuris*	Lythraceae	-	R/FR ratio, blue light intensity	[46]
*Sagittaria sagittifolia*	Alismataceae	arrowhead	light intensity	[19]
